# Characteristics and Causes for Non-Accrued Clinical Research (NACR) at an Academic Medical Institution

**DOI:** 10.4021/jocmr1320w

**Published:** 2013-04-23

**Authors:** Debra G. Tice, Kelly A. Carroll, Karishma H. Bhatt, Steven M. Belknap, David Mai, Heather J. Gipson, Dennis P. West

**Affiliations:** a Northwestern University, Institutional Review Board Office, 750 N. Lake Shore Drive, Room 725I, Chicago, IL 60611, USA; b Northwestern University, NU Clinical and Translational Sciences Institute, Feinberg School of Medicine, Chicago, IL 60611, USA; c Northwestern University, Feinberg School of Medicine, Department of Dermatology, 676 N. St. Clair St., Suite 1600, Chicago, IL 60611, USA; d Northwestern University, Feinberg School of Medicine, Departments of Dermatology and Medicine, 676 N. St. Clair St., Suite 1600, Chicago, IL 60611, USA; e Northwestern University, Feinberg School of Medicine, Departments of Dermatology and Pediatrics, 676 N. St. Clair St., Suite 1600, Chicago, IL 60611, USA

**Keywords:** Clinical research, Recruitment issues, Accrual

## Abstract

**Background:**

The impact of non-accrued clinical research (NACR) represents an important economic burden that is under consideration as the U.S. Department of Health and Human Services looks into reforming the regulations governing IRB review. NACR refers to clinical research projects that fail to enroll subjects. A delineation of the issues surrounding NACR is expected to enhance subject accrual and to minimize occurrence of NACR. The authors assessed demographics, characteristics, and reasons for NACR at an academic medical center, including time trends, funding source, research team (principal investigator, department), IRB resource utilization (IRB level of review, number of required IRB reviews, initial IRB turn-around time, and duration of NACR).

**Methods:**

The authors analyzed data from 848 clinical research study closures during 2010 and 2011 to determine proportion, incidence, and characteristics of NACR. Studies with subject enrollment during the same time period were used as a comparative measure.

**Results:**

Data from 704 (83.0%) study closures reported enrollment of 1 or more subjects while 144 (17.0 %) reported NACR (zero enrollment). PI-reported reasons for NACR included: 32 (22.2%) contract or funding issues; 43 (30.0%) insufficient study-dedicated resources; 41 (28.4%) recruitment issues; 17 (11.8%) sponsor-initiated study closure and 11 (7.6%) were “other/reason unreported”.

**Conclusions:**

NACR is not uncommon, affecting about one in six clinical research projects in the study population and reported to be more common in some other institutions. The complex and fluid nature of research conduct, non-realistic enrollment goals, and delays in both the approval and/or accrual processes contribute to NACR. Results suggest some simple strategies that investigators and institutions may use to reduce NACR, including careful feasibility assessment, reduction of institutional delays, and prompt initiation of subject accrual for multi-center studies using competitive enrollment. Institutional action to support investigators in the conduct clinical research is also encouraged to reduce likelihood of NACR.

## Introduction

NACR has not been adequately studied and the attendant financial burden to the institution has not been well characterized. NACR is defined in this report as an IRB-approved study reporting zero accrual at study closure. Accrued clinical research (ACR) was defined as an IRB-approved study that achieved enrollment of at least one subject. NACR wastes institutional resources and reduces IRB efficiency by competing for IRB review and approval with ACR. The authors sought to characterize the demographics and to identify proximate and ultimate causes of NACR.

## Materials and Methods

The analysis includes all human subject clinical research studies IRB-approved for closure at Northwestern University (NU) during calendar years, 2010 and 2011.

For each closed study, the following data was collected: funding source, PI name, department name, IRB level of review at initial approval, total number of both consenting and non-consenting studies, IRB submissions per study, initial turn-around time (time from IRB submission to IRB notification of approval in calendar days), study duration (time from IRB notification of approval to IRB closure in months) and reason for study closure as stated by the PI.

Clinical research studies were categorized based on the primary funding source for each study, namely; sponsored studies: (1) commercially-funded studies (private corporations), (2) federally-funded studies (federal-and/or state-supported), and (3) foundation-funded studies (from other agencies such as foundations or gifts from anonymous supporters) vs. non-sponsored studies (received institutional financial support) (Table 1).

**Table 1:** IRB Level of Review and Sponsorship Category for Both NACR and ACR, Northwestern University, 2010 - 2011

	NACR			ACR			Total

Sponsorship	NACR	Expedited Review	Full Panel Review	ACR	Expedited Review	Full Panel Review	NACR & ACR (% of Total)
Sponsored Studies							
Industry-funded	60	6	54	212	35	177	272 (32.0%)
Federally-funded	30	9	21	140	63	77	170 (20.0%)
Foundation-funded	13	8	5	55	36	19	68 (8.0%)
Total Sponsored Studies	103	23	80	407	134	273	510 (60.0%)

Non-Sponsored Studies							
Total Non-Sponsored Studies	41	27	14	297	239	58	338 (40.0%)

Total	144	50	94	704	373	331	848 (100.0%)

Number of Studies includes projects that required written or verbal consent as well as those that have waived consent

When comparing the rate of NACR to ACR in sponsored vs. non-sponsored studies the attributable risk for NACR = 0.081, (CI = 0.031 - 0.130, P = 0.0021, Fishers Exact Test).

Data collected for each NACR study included: the number of IRB convened panels and expedited reviews of all types (for example, initial new project reviews, revisions, annual reviews).

Reasons for NACR were categorized based on the PI-stated reason in the IRB closure submission. Where relevant, the PI-designated reason for NACR was verified by reviewing other documents in the IRB closure submission, including sponsor documentation.

## Results

### Relevant trends of NACR

During calendar years 2010 and 2011, the institution’s IRB approved 848 study closure submissions, of which 704 (83.0%) reported ACR and 144 (17.0%) reported NACR.

### Sponsorship

For NACR, 103 (71.5%) were sponsored; for ACR 407 (60%) were sponsored (Table 1). The attributable risk of NACR in sponsored vs. non-sponsored studies was AR = 0.081, (CI - 0.031 - 0.130, P = 0.0021, Fishers Exact Test).

### PI/Department

NACR was not uniformly distributed among PIs and/or departments. A total of 328 PIs closed 848 studies. Closure data by PI (Table 2) shows that 224 PIs (68.3%) reported no NACR while 33 PIs (10%) had only NACR; the remaining 71 PIs (21.7%) had at least one NACR study.

**Table 2:** Summary of IRB Study Closures According to Reporting Entity, Northwestern University, 2010 - 2011

Reporting Entity	No NACR	≤ 1 NACR	Only NACR	Total
Principal Investigator	224 (68.3%)	71 (21.7%)	33 (10.0%)	328
Department/ Division	31 (42.3%)	34 (48.6)	5 (7.1%)	70

For 70 departments that represented 848 closed studies (Table 2), 42.3% reported ACR compared to 57.7% that reported at least one NACR study, with five (7.1% of departments) having only NACR. One department had more than 10 NACR studies, 24 departments had 2�C10 NACR studies.

### IRB resource utilization

#### Categorization of IRB level of review

Of the 144 NACR study closures, 94 (65.3%) studies underwent initial IRB review by a convened panel. Of the 704 closures with “enrolled” subjects, 331 (47.0%) studies underwent initial IRB review by a convened panel and 373 (53.0%) were reviewed expeditedly (some of which were non-consenting chart reviews or tissue analysis). Of note, expedited studies correlated with less NACR (Table 1).

#### Number of IRB reviews

In addition to 144 new study submissions resulting in NACR, these same NACR studies generated 607 subsequent IRB submissions (such as annual reviews, safety reports and revisions) (Tables 3, 4).

**Table 3:** Total Number of Full Panel Reviews for 94 NACR Studies According to Submission Type and Sponsorship, Northwestern University, 2010 - 2011

	Submission Type (# of deferred submissions)				

Sponsorship	New Project	Continuing Review	Revision	Safety-Other	Total
Industry-funded	54 (10)	19 (1)	35	11	119 (11)
Federally-funded	21 (4)	4	5	2	32 (4)
Foundation-funded	5 (1)	0	0	0	5 (1)
Non-Sponsored Studies	14 (8)	1	3 (3)	0	18 (11)
Total	94 (23)	24 (1)	43 (3)	13	174 (27)

This includes studies that require written or verbal consent as well as projects that have waived consent.

**Table 4:** Total Number of Expedited Reviews for 50 NACR Studies According to Submission Type and Sponsorship Northwestern University, 2010 - 2011

	Submission Type (# of deferred submissions)					

Sponsorship	New Project	Continuing Review	Revision	Safety-Other	Termination	Total
Industry-funded	6	19	129	12	60	222
Federally-funded	9	34	57	0	30	131
Foundation-funded	8	8	10	0	13	39
Non-Sponsored Studies	27	55	58	1	41	182
Total	50	116	254	13	144	577

This includes studies that require written or verbal consent as well as projects that have waived consent.

Note that the convened IRB panel “deferred” 23 (24.5%) of the 94 new study submissions at initial IRB panel review, requiring subsequent additional panel reviews. Six of the new study submissions were deferred twice before obtaining IRB approval.

Of interest, Industry-sponsored studies represented a majority of the IRB subsequent submissions (Tables 3, 4) and submission of new safety information or protocol violations occurred most frequently for industry-funded studies.

Unsurprisingly, non-sponsored studies remained active at the IRB for a longer period of time compared to sponsored studies; sponsored mean duration = 18.3 months (range 1.7 - 70.7, median 14.0) months compared to non-sponsored mean duration = 27.0 months (range 5.3 - 78.2, median 22.5).

#### IRB metrics

The mean initial turn-around time (number of calendar days between IRB submission to notification of IRB approval) for expedited new studies was 51 days (range 0 - 341) and the initial turn-around time for convened panel-reviewed new studies was 60 days (range 16 - 211). Variations were identified according to type of funding source and IRB level of review. For all new studies, the mean turn-around time was significantly longer at 70.8 days for non-sponsored vs. 51 days for sponsored studies (P = 0.0195, two-tailed, two sample t-test assuming equal variances).

From IRB approval to closure, NACR studies were open a mean of 20.8 months (range 1.7 - 78.2, median = 18) while ACR studies were open significantly longer with a mean of 42.9 months (range 2.7 - 233.8, median = 34.5). There was variation according to type of sponsorship with industry-sponsored studies opened a mean of 15.7 months, (range 1.7 - 44.2, median = 10.8 months) and non-sponsored studies opened a mean of 27 months range 5.3 - 78.2, median = 22.5). Of note, eight (8) of these 144 studies resulting in NACR were open at the IRB for more than 4 years and 7 of the 8 were non-sponsored studies.

### Reasons for NACR

**Figure 1:**
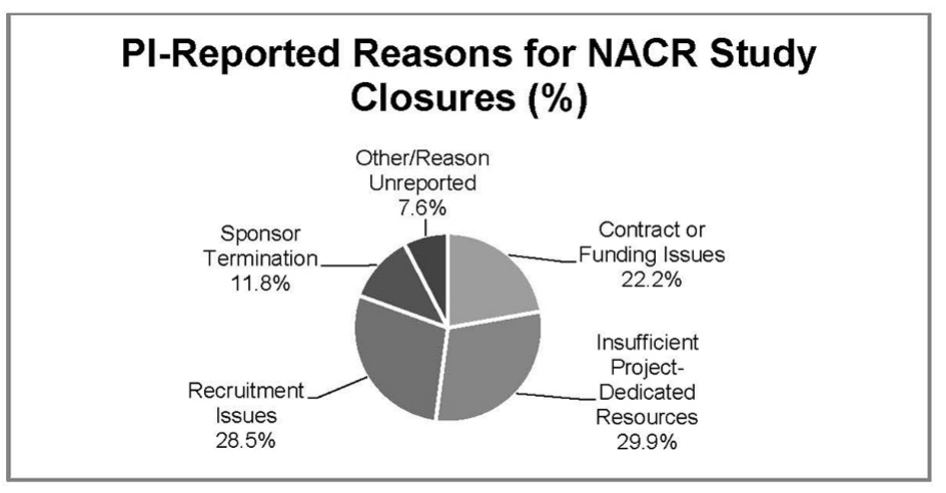
PI reported reasons for biomedical NACR study closures Northwestern University, 2010 - 2011.

While Figure 1 provides an overview of how frequently a PI-stated reason was associated with NACR, a detailed review of PI-stated reasons for closure by type of sponsorship is provided (Table 5). The most common PI-reported reason for closure is Insufficient study-dedicated resources, followed closely by Recruitment issues at 28.4%. Within Insufficient study-dedicated resources, the most common stated reason (18) is Other priorities. Of the 41 studies for which the PI reported Recruitment issues as the reason for NACR, Sponsor had already met enrollment accounted for the majority. Interestingly, NACR associated with both Contract issues and Funding issues (sponsored and non-sponsored) represented 22% of total NACR.

**Table 5:** PI-Reported Reasons for NACR by Sponsorship Categories (Number of Studies), Northwestern University, 2010 - 2011

Reason for NACR	Non-Sponsored	Industry-Funded	Federally-Funded	Foundation-Funded	Total (% of NACR)
Insufficient Study-Dedicated Resources (n = 43)	28	4	8	3	43 (30.0%)
Recruitment Issues (n = 41)	2	30	9	0	41 (28.4%)
Contract of Funding Issues (n = 32)	6	11	9	6	32 (22.2%)
Sponsor Termination (n = -17)	2	11	3	1	17 (11.8%)
Other/Reason Unreported (n = 11)	3	4	1	3	11 (7.6%)
Total	41	60	30	13	144 (100.0%)

Relationship to NACR differs according to the type of study sponsorship. Within non-sponsored studies, the most commonly stated reason was Insufficient study-dedicated resources. For Sponsored studies, Recruitment issues were the most commonly stated reason for NACR.

Notably, Recruitment issues accounted for half of industry - funded NACR stated reasons, with most of those issues being related to Sponsor met enrollment. (namely, the total enrollment goal for a multi-center study was met prior to enrollment of a single subject at this site).

Unlike Industry funded studies, where Contract issues stood out, in Federally-funded studies Funding issues were more commonly noted as a reason for NACR. Interestingly, Sponsor had already met enrollment was the most commonly stated reason for Recruitment issues in both Industry and multi-centered Federally-funded studies.

## Discussion

The federal regulations mandate that an institutional review board independently review and approve all non-exempt human subject research before a study may begin. While there is sufficient evidence that IRB review is costly and may account for some of the delays in project initiation of a research study, research productivity should be tied to the number of clinical research studies that meet accrual goals and avoid NACR, not simple the number of currently approved IRB studies and/or award dollar amounts [1].

This report is novel for two important reasons. First, the data represent all clinical research studies and are not limited to specific study populations. Second, this report addresses causality by addressing the study-specific PI-state reason. Kitterman et al also conducted a retrospective survey to identify barriers prior to/after study opening to identify reasons for low-enrollment [2]. It was observed that the majority of the barriers occurred after the initiation of the study (namely rare diseases, epidemiology/tissue collection studies and delays from the FDA as key barriers after study initiation). One Clinical & Translational Science Award (CTSA) academic medical center, in their analysis of IRB studies terminated between FY 2005 and 2009, identified 260 out of 837 (31.1%) as zero or low-enrolling studies (0-1 subject) [3]. The economic impact of this low enrollment for studies closed in FY 2009 was estimated to be approximately $1 million. Other CTSA academic institutions have recently reported that 20-47% of clinical trial studies failed to enroll subjects [4-6]. The National Cancer Institute (NCI) cooperative group terminated up to 45% of phase III trials due to accrual being too low to meet scientific objectives [7-11].

Insufficient study-dedicated resources was the most frequent PI-attributed causality for NACR, despite endorsement by the investigators and departmental chair that sufficient internal resources (properly trained staff and adequate study facilities and equipment) were available to adequately conduct the research as proposed and that the proposal has been reviewed for scientific merit or other department-specific requirements prior to IRB approval. A majority of studies in this category were non-sponsored.

It seems plausible that conduct of a full scientific or resource-related feasibility assessment would lower the rate of NACR but data from federally-designated Comprehensive Cancer centers across the nation, where such reviews are conducted in a standardized manner by panels, reveal that a relatively high rate of NACR and low enrolling ACR or NACR (up to 17% of studies) exist [7-11].

Recruitment Issues was the second most commonly attributed reason for NACR. Those involved in the clinical research enterprise generally agree that subject recruitment is a significant potential cause for delay in the clinical trial process [12, 13]. Due to real costs associated with poor accrual as well as identification of costly sites that do not perform, improvement in the site selection process has been estimated to avoid $6 billion a year in lost revenues for the pharmaceutical industry alone [14, 15].

### Strategies to reduce NACR

Several strategies may be directed to address slow accrual. A feasibility assessment of the performance site, routinely performed by industry sponsors, mostly involves self-reporting by the PI on past performance as well as site characteristics and performance on previous trials. Questions may probe for previous accrual and retention rates as well as for data quality (rate of data queries and resolutions) for the site. This assessment also seeks to determine the proposed recruitment strategy, including number of potential subjects at the study site. Sponsors prefer objective data - the type of data obtained from an electronic database - that accurately reflects the number of patients that match the entry criteria. These feasibility assessments often do not require institutional review or endorsement. In essence, a PI can often self-report site characteristics of the study site and performance history with little to no institutional oversight.

### Feasibility assessments

Institutional feasibility assessments of clinical research studies need to be conducted and must address both scientific merit as well as strong recruitment potential prior to submission to the IRB. This should improve efficiency of the IRB approval process as well as accomplish improved subject accrual. While the cost and efficiency savings may be obvious, what is often overlooked is the cost to human subjects who participate in inadequately accruing studies. While the institution bears the cost for NACR, it is the subject who has been placed at risk with limited, if any, possibility for future benefit when a study does not meet its accrual goal.

To address this, local feasibility assessments, whether conducted by the Department or the PI, should include detailed and specific questions about accrual goals [15]. Recruitment strategy may often include an electronic search, if applicable, whether via a resource such as electronic medical records (EMRs) or disease related registries as part of the feasibility assessment. The assessment should also include a review of competing studies [14]. An essential question is, how many other studies are currently open that will be recruiting from the same subject population?

### Recruitment methods

A second strategy to address accrual goal is the development of a realistic recruitment strategy that is regularly evaluated for outcome. Although there are effective strategies for recruiting subjects to clinical research, it is not always clear which strategy best targets the study population to identify candidates for participation [13, 16-18]. Developing a clear, multi-tiered plan that includes both a recruitment budget and/or resources for recruitment, as well as a plan for assessing accrual rate is essential to successful accrual. PIs who continue to meet their recruitment goals tend to follow a similar approach for each study [15]. Single tiered plans that rely on a narrowly based strategy have few options for meeting accrual goals if the initial method does not meet these goals. Reliance on the PI finding subjects in their clinical practice or from a research registry may not be broad-based enough or well-targeted to the study population and therefore budgeting for recruitment strategy is essential to meeting accrual goals. Tracking the success of the recruitment plan at the Department or Institutional level provides critical information on recruitment strategies that are or are not working so that timely adjustments can be made to recruitment methodology.

Finally, how can the institution help potential candidate subjects find research studies in which to participate? It is estimated that 76% of patients express a desire to enroll in clinical trials when they have sufficient information available to them [19]. Eighty percent of Americans report satisfactory results when searching for health information on the Internet. When searching for clinical trial information, on the other hand, individuals report that it is not user-friendly to navigate websites to identify possible trials (for example, ClinicalTrials.gov) [20]. Presenting clinical trials information that is understandable to the consumer and that “is suited to their information seeking behavior” is critical to increasing the identification of potential candidate subjects for a research study [14]. Instead of multiple independent silos, by developing a centralized, user-friendly recruitment center for the institution, subjects should be able to easily find studies and express their interest to participate [21].

### Institutional support

A third strategy for addressing accrual goals is institutionally based. It involves providing tools that can be easily, effectively, and inexpensively accessed by investigators. One such tool is integration of EMRs [22]. EMRs may pool clinical data from hospital and physician practices associated with the Institution. Integrated EMRs can provide the number of subjects who meet specific study criteria and can be used to identify prospective subjects to contact about research participation. As with such systems, there are also limitations: 1) costs may not be negligible and may be allocated to the Department; 2) the medical record data that exist in EMRs may not be granular to some specific inclusion/exclusion criteria [23]; 3) not all care providers may utilize a medical record system that is within the integrated EMRs; and 4) such EMR systems most commonly are unidirectional from care provider to patient, which means that patients often cannot search for research opportunities in such a system [14, 23].

A second useful institutional tool is a research registry. This is a rich resource at many academic medical centers with numerous department-based IRB-approved research registries. These research databases assist IRB-approved investigators in finding potential subjects [22]. They are frequently updated and lead to persons who have expressed an interest in being contacted about participating in research. Unfortunately, these registries are not well publicized and there is a considerable cost associated with maintaining research registries.

Contract or funding issues were the next most attributed reasons for NACR. IRB review and approval often must be completed before full execution of a contract. PIs might consider the following recommendations when submitting IRB projects that are undergoing contract or funding issues: (1) Submit sponsored studies to the IRB for review after the project has a finalized protocol and the site has been chosen and considered fundable. (2) For sponsored studies there is often a gap between IRB submission date and the date of submission to the grants and contracts office. It is estimated that the asynchronous submission process adds more than 30 days (approximately 20%) to clinical trial approvals [24].

### IRB solutions

An in-house survey of the research community ranked “timeliness of reviews” as the #1 priority [25]. There has been a great deal written on how to improve the efficiency of IRB review but there are two potential, readily-reorganized steps to decrease turn-around time of IRB submissions: 1). Conduct Pre-review of complex studies by specially trained departmental staff, those familiar with regulatory requirements and study risks of the study before submission to the IRB Office. This pre-review could serve as a quality check on the submission to detect and correct errors that would otherwise delay the review process and result IRB deferral decisions as well as unnecessary efforts by both the IRB and the PI. 2). Academic-based local IRBs should incorporate some aspects of the centralized IRB model to achieve more prompt review and determination. Frequent IRB panel meetings effectively meet the needs of the PI and the sponsor. In certain circumstances, with IRB oversight there may be adequate justification for combined streamlined central and local IRB reviews utilizing an IAA (IRB Authorization Agreement) and an expert reviewer under Facilitated Review of centrally IRB-approved biomedical projects.

### Conclusions

NACR negatively impacts the institution with its attendant undue economic burden related to poor resource utilization. This report details some of the characteristics and PI- stated reasons for NACR as well as possible approaches to resolution.

## Previous Presentations

Partial results of this study were presented at the Public Responsibility in Medicine and Research (PRIMR&R) 2011 Advancing Ethical Research Conference, 12/4/2012, San Diego, California.

Partial results of this study were presented to the 2012 5th Annual Clinical and Translational Science Award (CTSA) Clinical Research Management Workshop, 6/4/2012, Yale University, New Haven, Connecticut.
